# The global distribution of lymphatic filariasis, 2000–18: a geospatial analysis

**DOI:** 10.1016/S2214-109X(20)30286-2

**Published:** 2020-08-19

**Authors:** Local Burden of Disease 2019 Neglected Tropical Diseases Collaborators, Aniruddha Deshpande, Molly K Miller-Petrie, Paulina A Lindstedt, Mathew M Baumann, Kimberly B Johnson, Brigette F Blacker, Hedayat Abbastabar, Foad Abd-Allah, Ahmed Abdelalim, Ibrahim Abdollahpour, Kedir Hussein Abegaz, Ayenew Negesse Abejie, Lucas Guimarães Abreu, Michael R.M. Abrigo, Ahmed Abualhasan, Manfred Mario Kokou Accrombessi, Abdu A Adamu, Oladimeji M Adebayo, Isaac Akinkunmi Adedeji, Rufus Adesoji Adedoyin, Victor Adekanmbi, Olatunji O Adetokunboh, Tara Ballav Adhikari, Mohsen Afarideh, Marcela Agudelo-Botero, Mehdi Ahmadi, Keivan Ahmadi, Muktar Beshir Ahmed, Anwar E Ahmed, Temesgen Yihunie Akalu, Ali S Akanda, Fares Alahdab, Ziyad Al-Aly, Samiah Alam, Noore Alam, Genet Melak Alamene, Turki M Alanzi, James Albright, Ammar Albujeer, Jacqueline Elizabeth Alcalde-Rabanal, Animut Alebel, Zewdie Aderaw Alemu, Muhammad Ali, Mehran Alijanzadeh, Vahid Alipour, Syed Mohamed Aljunid, Ali Almasi, Amir Almasi-Hashiani, Hesham M Al-Mekhlafi, Khalid A Altirkawi, Nelson Alvis-Guzman, Nelson J. Alvis-Zakzuk, Saeed Amini, Arianna Maever L. Amit, Gianna Gayle Herrera Amul, Catalina Liliana Andrei, Mina Anjomshoa, Ansariadi Ansariadi, Carl Abelardo T. Antonio, Benny Antony, Ernoiz Antriyandarti, Jalal Arabloo, Hany Mohamed Amin Aref, Olatunde Aremu, Bahram Armoon, Amit Arora, Krishna K Aryal, Afsaneh Arzani, Mehran Asadi-Aliabadi, Daniel Asmelash, Hagos Tasew Atalay, Seyyede Masoume Athari, Seyyed Shamsadin Athari, Sachin R Atre, Marcel Ausloos, Shally Awasthi, Nefsu Awoke, Beatriz Paulina Ayala Quintanilla, Getinet Ayano, Martin Amogre Ayanore, Yared Asmare Aynalem, Samad Azari, Andrew S Azman, Ebrahim Babaee, Alaa Badawi, Mojtaba Bagherzadeh, Shankar M Bakkannavar, Senthilkumar Balakrishnan, Maciej Banach, Joseph Adel Mattar Banoub, Aleksandra Barac, Miguel A Barboza, Till Winfried Bärnighausen, Sanjay Basu, Vo Dinh Bay, Mohsen Bayati, Neeraj Bedi, Mahya Beheshti, Meysam Behzadifar, Masoud Behzadifar, Diana Fernanda Bejarano Ramirez, Michelle L Bell, Derrick A. Bennett, Habib Benzian, Dessalegn Ajema Berbada, Robert S Bernstein, Anusha Ganapati Bhat, Krittika Bhattacharyya, Soumyadeep Bhaumik, Zulfiqar A Bhutta, Ali Bijani, Boris Bikbov, Muhammad Shahdaat Bin Sayeed, Raaj Kishore Biswas, Somayeh Bohlouli, Soufiane Boufous, Oliver J Brady, Andrey Nikolaevich Briko, Nikolay Ivanovich Briko, Gabrielle B Britton, Alexandria Brown, Sharath Burugina Nagaraja, Zahid A Butt, Luis Alberto Cámera, Ismael R Campos-Nonato, Julio Cesar Campuzano Rincon, Jorge Cano, Josip Car, Rosario Cárdenas, Felix Carvalho, Carlos A Castañeda-Orjuela, Franz Castro, Ester Cerin, Binaya Chalise, Vijay Kumar Chattu, Ken Lee Chin, Devasahayam J Christopher, Dinh-Toi Chu, Natalie Maria Cormier, Vera Marisa Costa, Elizabeth A Cromwell, Abel Fekadu Fekadu Dadi, Tukur Dahiru, Saad M A Dahlawi, Rakhi Dandona, Lalit Dandona, Anh Kim Dang, Farah Daoud, Aso Mohammad Darwesh, Amira Hamed Darwish, Ahmad Daryani, Jai K Das, Rajat Das Gupta, Aditya Prasad Dash, Claudio Alberto Dávila-Cervantes, Nicole Davis Weaver, Fernando Pio De la Hoz, Jan-Walter De Neve, Dereje Bayissa Demissie, Gebre Teklemariam Demoz, Edgar Denova-Gutiérrez, Kebede Deribe, Assefa Desalew, Samath Dhamminda Dharmaratne, Preeti Dhillon, Meghnath Dhimal, Govinda Prasad Dhungana, Daniel Diaz, Isaac Oluwafemi Dipeolu, Hoa Thi Do, Christiane Dolecek, Kerrie E Doyle, Eleonora Dubljanin, Andre Rodrigues Duraes, Hisham Atan Edinur, Andem Effiong, Aziz Eftekhari, Nevine El Nahas, Maysaa El Sayed Zaki, Maha El Tantawi, Hala Rashad Elhabashy, Shaimaa I. El-Jaafary, Ziad El-Khatib, Hajer Elkout, Aisha Elsharkawy, Shymaa Enany, Daniel Adane Endalew, Babak Eshrati, Sharareh Eskandarieh, Arash Etemadi, Oluchi Ezekannagha, Emerito Jose A. Faraon, Mohammad Fareed, Andre Faro, Farshad Farzadfar, Alebachew Fasil Fasil, Mehdi Fazlzadeh, Valery L. Feigin, Wubalem Fekadu, Netsanet Fentahun, Seyed-Mohammad Fereshtehnejad, Eduarda Fernandes, Irina Filip, Florian Fischer, Carsten Flohr, Nataliya A. Foigt, Morenike Oluwatoyin Folayan, Masoud Foroutan, Richard Charles Franklin, Joseph Jon Frostad, Takeshi Fukumoto, Mohamed M Gad, Gregory M Garcia, Augustine Mwangi Gatotoh, Reta Tsegaye Gayesa, Ketema Bizuwork Gebremedhin, Yilma Chisha Dea Geramo, Hailay Abrha Gesesew, Kebede Embaye Gezae, Ahmad Ghashghaee, Farzaneh Ghazi Sherbaf, Tiffany K Gill, Paramjit Singh Gill, Themba G Ginindza, Alem Girmay, Zemichael Gizaw, Amador Goodridge, Sameer Vali Gopalani, Bárbara Niegia Garcia Goulart, Alessandra C Goulart, Ayman Grada, Manfred S Green, Mohammed Ibrahim Mohialdeen Gubari, Harish Chander Gugnani, Davide Guido, Rafael Alves Guimarães, Yuming Guo, Rajeev Gupta, Rahul Gupta, Giang Hai Ha, Juanita A. Haagsma, Nima Hafezi-Nejad, Dessalegn H Haile, Michael Tamene Haile, Brian J. Hall, Samer Hamidi, Demelash Woldeyohannes Handiso, Hamidreza Haririan, Ninuk Hariyani, Ahmed I. Hasaballah, Md. Mehedi Hasan, Amir Hasanzadeh, Hamid Yimam Hassen, Desta Haftu Hayelom, Mohamed I Hegazy, Behzad Heibati, Behnam Heidari, Delia Hendrie, Andualem Henok, Claudiu Herteliu, Fatemeh Heydarpour, Hagos Degefa de Hidru, Thomas R Hird, Chi Linh Hoang, Gillian I Hollerich, Praveen Hoogar, Naznin Hossain, Mehdi Hosseinzadeh, Mowafa Househ, Guoqing Hu, Ayesha Humayun, Syed Ather Hussain, Mamusha Aman A Hussen, Segun Emmanuel Ibitoye, Olayinka Stephen Ilesanmi, Milena D. Ilic, Mohammad Hasan Imani-Nasab, Usman Iqbal, Seyed Sina Naghibi Irvani, Sheikh Mohammed Shariful Islam, Rebecca Q Ivers, Chinwe Juliana Iwu, Nader Jahanmehr, Mihajlo Jakovljevic, Amir Jalali, Achala Upendra Jayatilleke, Ensiyeh Jenabi, Ravi Prakash Jha, Vivekanand Jha, John S Ji, Jost B. Jonas, Jacek Jerzy Jozwiak, Ali Kabir, Zubair Kabir, Tanuj Kanchan, André Karch, Surendra Karki, Amir Kasaeian, Gebremicheal Gebreslassie Kasahun, Habtamu Kebebe Kasaye, Gebrehiwot G Kassa, Getachew Mullu Kassa, Gbenga A. Kayode, Mihiretu M Kebede, Peter Njenga Keiyoro, Daniel Bekele Ketema, Yousef Saleh Khader, Morteza Abdullatif Khafaie, Nauman Khalid, Rovshan Khalilov, Ejaz Ahmad Khan, Junaid Khan, Md Nuruzzaman Khan, Khaled Khatab, Mona M Khater, Amir M Khater, Maryam Khayamzadeh, Mohammad Khazaei, Mohammad Hossein Khosravi, Jagdish Khubchandani, Ali Kiadaliri, Yun Jin Kim, Ruth W Kimokoti, Sezer Kisa, Adnan Kisa, Sonali Kochhar, Tufa Kolola, Hamidreza Komaki, Soewarta Kosen, Parvaiz A Koul, Ai Koyanagi, Kewal Krishan, Barthelemy Kuate Defo, Nuworza Kugbey, Pushpendra Kumar, G Anil Kumar, Manasi Kumar, Dian Kusuma, Carlo La Vecchia, Ben Lacey, Aparna Lal, Dharmesh Kumar Lal, Hilton Lam, Faris Hasan Lami, Van Charles Lansingh, Savita Lasrado, Georgy Lebedev, Paul H Lee, Kate E LeGrand, Mostafa Leili, Tsegaye Lolaso Lenjebo, Cheru Tesema Leshargie, Aubrey J Levine, Sonia Lewycka, Shanshan Li, Shai Linn, Shiwei Liu, Jaifred Christian F Lopez, Platon D Lopukhov, Muhammed Magdy Abd El Razek, D.R. Mahadeshwara Prasad, Phetole Walter Mahasha, Narayan B. Mahotra, Azeem Majeed, Reza Malekzadeh, Deborah Carvalho Malta, Abdullah A Mamun, Navid Manafi, Mohammad Ali Mansournia, Chabila Christopher Mapoma, Gabriel Martinez, Santi Martini, Francisco Rogerlândio Martins-Melo, Manu Raj Mathur, Benjamin K Mayala, Mohsen Mazidi, Colm McAlinden, Birhanu Geta Meharie, Man Mohan Mehndiratta, Entezar Mehrabi Nasab, Kala M Mehta, Teferi Mekonnen, Tefera Chane Mekonnen, Gebrekiros Gebremichael Meles, Hagazi Gebre Meles, Peter T N Memiah, Ziad A Memish, Walter Mendoza, Ritesh G Menezes, Seid Tiku Mereta, Tuomo J Meretoja, Tomislav Mestrovic, Workua Mekonnen Metekiya, Workua Mekonnen Metekiya, Bartosz Miazgowski, Ted R Miller, GK Mini, Erkin M Mirrakhimov, Babak Moazen, Bahram Mohajer, Yousef Mohammad, Dara K. Mohammad, Naser Mohammad Gholi Mezerji, Roghayeh Mohammadibakhsh, Shafiu Mohammed, Jemal Abdu Mohammed, Hassen Mohammed, Farnam Mohebi, Ali H Mokdad, Yoshan Moodley, Masoud Moradi, Ghobad Moradi, Mohammad Moradi-Joo, Paula Moraga, Linda Morales, Abbas Mosapour, Jonathan F. Mosser, Simin Mouodi, Seyyed Meysam Mousavi, Miliva Mozaffor, Sandra B Munro, Moses K. Muriithi, Christopher J L Murray, Kamarul Imran Musa, Ghulam Mustafa, Saravanan Muthupandian, Mehdi Naderi, Ahamarshan Jayaraman Nagarajan, Mohsen Naghavi, Gurudatta Naik, Vinay Nangia, Bruno Ramos Nascimento, Javad Nazari, Duduzile Edith Ndwandwe, Ionut Negoi, Henok Biresaw Netsere, Josephine W. Ngunjiri, Cuong Tat Nguyen, Huong Lan Thi Nguyen, QuynhAnh P Nguyen, Solomon Gedlu Nigatu, Dina Nur Anggraini Ningrum, Chukwudi A Nnaji, Marzieh Nojomi, Ole F Norheim, Jean Jacques Noubiap, Bogdan Oancea, Felix Akpojene Ogbo, In-Hwan Oh, Andrew T Olagunju, Jacob Olusegun Olusanya, Bolajoko Olubukunola Olusanya, Obinna E Onwujekwe, Doris V. Ortega-Altamirano, Osayomwanbo Osarenotor, Frank B Osei, Mayowa O Owolabi, Mahesh P A, Jagadish Rao. Padubidri, Smita Pakhale, Adrian Pana, Eun-Kee Park, Sangram Kishor Patel, Ashish Pathak, Ajay Patle, Kebreab Paulos, Veincent Christian Filipino Pepito, Norberto Perico, Aslam Pervaiz, Julia Moreira Pescarini, Konrad Pesudovs, Hai Quang Pham, David M Pigott, Thomas Pilgrim, Meghdad Pirsaheb, Mario Poljak, Ian Pollock, Maarten J Postma, Farshad Pourmalek, Akram Pourshams, Sergio I Prada, Liliana Preotescu, Hedley Quintana, Navid Rabiee, Mohammad Rabiee, Amir Radfar, Alireza Rafiei, Fakher Rahim, Siavash Rahimi, Vafa Rahimi-Movaghar, Muhammad Aziz Rahman, Mohammad Hifz Ur Rahman, Fatemeh Rajati, Chhabi Lal Ranabhat, Puja C Rao, Davide Rasella, Goura Kishor Rath, Salman Rawaf, Lal Rawal, Wasiq Faraz Rawasia, Giuseppe Remuzzi, Vishnu Renjith, Andre M.N. Renzaho, Serge Resnikoff, Seyed Mohammad Riahi, Ana Isabel Ribeiro, Jennifer Rickard, Leonardo Roever, Luca Ronfani, Enrico Rubagotti, Salvatore Rubino, Anas M Saad, Siamak Sabour, Ehsan Sadeghi, Sahar Saeedi Moghaddam, Yahya Safari, Rajesh Sagar, Mohammad Ali Sahraian, S. Mohammad Sajadi, Mohammad Reza Salahshoor, Nasir Salam, Ahsan Saleem, Hosni Salem, Marwa Rashad Salem, Yahya Salimi, Hamideh Salimzadeh, Abdallah M Samy, Juan Sanabria, Itamar S Santos, Milena M. Santric-Milicevic, Bruno Piassi Sao Jose, Sivan Yegnanarayana Iyer Saraswathy, Nizal Sarrafzadegan, Benn Sartorius, Brijesh Sathian, Thirunavukkarasu Sathish, Maheswar Satpathy, Monika Sawhney, Mehdi Sayyah, Alyssa N Sbarra, Lauren E Schaeffer, David C Schwebel, Anbissa Muleta Senbeta, Subramanian Senthilkumaran, Sadaf G Sepanlou, Edson Serván-Mori, Azadeh Shafieesabet, Amira A Shaheen, Izza Shahid, Masood Ali Shaikh, Ali S Shalash, Mehran Shams-Beyranvand, MohammadBagher Shamsi, Morteza Shamsizadeh, Mohammed Shannawaz, Kiomars Sharafi, Rajesh Sharma, Aziz Sheikh, B Suresh Kumar Shetty, Wondimeneh Shibabaw Shiferaw, Mika Shigematsu, Jae Il Shin, Rahman Shiri, Reza Shirkoohi, K M Shivakumar, Si Si, Soraya Siabani, Tariq Jamal Siddiqi, Diego Augusto Santos Silva, Virendra Singh, Narinder Pal Singh, Balbir Bagicha Singh Singh, Jasvinder A. Singh, Ambrish Singh, Dhirendra Narain Sinha, Malede Mequanent Sisay, Eirini Skiadaresi, David L Smith, Adauto Martins Soares Filho, Mohammad Reza Sobhiyeh, Anton Sokhan, Joan B Soriano, Muluken Bekele Sorrie, Ireneous N Soyiri, Emma Elizabeth Spurlock, Chandrashekhar T Sreeramareddy, Agus Sudaryanto, Mu'awiyyah Babale Sufiyan, Hafiz Ansar Rasul Suleria, Bryan L. Sykes, Rafael Tabarés-Seisdedos, Takahiro Tabuchi, Degena Bahrey Tadesse, Ingan Ukur Tarigan, Bineyam Taye, Yonatal Mesfin Tefera, Arash Tehrani-Banihashemi, Shishay Wahdey Tekelemedhin, Merhawi Gebremedhin Tekle, Mohamad-Hani Temsah, Berhe Etsay Tesfay, Fisaha Haile Tesfay, Zemenu Tadesse Tessema, Kavumpurathu Raman Thankappan, Akhil Soman ThekkePurakkal, Nihal Thomas, Robert L Thompson, Alan J Thomson, Roman Topor-Madry, Marcos Roberto Tovani-Palone, Eugenio Traini, Bach Xuan Tran, Khanh Bao Tran, Irfan Ullah, Bhaskaran Unnikrishnan, Muhammad Shariq Usman, Olalekan A Uthman, Benjamin S. Chudi Uzochukwu, Pascual R Valdez, Santosh Varughese, Yousef Veisani, Francesco S Violante, Sebastian Vollmer, Feleke Gebremeskel W/hawariat, Yasir Waheed, Mitchell Taylor Wallin, Yuan-Pang Wang, Yafeng Wang, Kinley Wangdi, Daniel J Weiss, Girmay Teklay Weldesamuel, Adhena Ayaliew Werkneh, Ronny Westerman, Taweewat Wiangkham, Kirsten E Wiens, Tissa Wijeratne, Charles Shey Wiysonge, Haileab Fekadu Wolde, Dawit Zewdu Wondafrash, Tewodros Eshete Wonde, Getasew Taddesse Worku, Ali Yadollahpour, Seyed Hossein Yahyazadeh Jabbari, Tomohide Yamada, Mehdi Yaseri, Hiroshi Yatsuya, Alex Yeshaneh, Mekdes Tigistu Yilma, Paul Yip, Engida Yisma, Naohiro Yonemoto, Mustafa Z Younis, Hebat-Allah Salah A Yousof, Chuanhua Yu, Hasan Yusefzadeh, Siddhesh Zadey, Telma Zahirian Moghadam, Zoubida Zaidi, Sojib Bin Zaman, Mohammad Zamani, Hamed Zandian, Heather J Zar, Taddese Alemu Zerfu, Yunquan Zhang, Arash Ziapour, Sanjay Zodpey, Yves Miel H Zuniga, Simon I Hay, Robert C Reiner

## Abstract

**Background:**

Lymphatic filariasis is a neglected tropical disease that can cause permanent disability through disruption of the lymphatic system. This disease is caused by parasitic filarial worms that are transmitted by mosquitos. Mass drug administration (MDA) of antihelmintics is recommended by WHO to eliminate lymphatic filariasis as a public health problem. This study aims to produce the first geospatial estimates of the global prevalence of lymphatic filariasis infection over time, to quantify progress towards elimination, and to identify geographical variation in distribution of infection.

**Methods:**

A global dataset of georeferenced surveyed locations was used to model annual 2000–18 lymphatic filariasis prevalence for 73 current or previously endemic countries. We applied Bayesian model-based geostatistics and time series methods to generate spatially continuous estimates of global all-age 2000–18 prevalence of lymphatic filariasis infection mapped at a resolution of 5 km^2^ and aggregated to estimate total number of individuals infected.

**Findings:**

We used 14 927 datapoints to fit the geospatial models. An estimated 199 million total individuals (95% uncertainty interval 174–234 million) worldwide were infected with lymphatic filariasis in 2000, with totals for WHO regions ranging from 3·1 million (1·6–5·7 million) in the region of the Americas to 107 million (91–134 million) in the South-East Asia region. By 2018, an estimated 51 million individuals (43–63 million) were infected. Broad declines in prevalence are observed globally, but focal areas in Africa and southeast Asia remain less likely to have attained infection prevalence thresholds proposed to achieve local elimination.

**Interpretation:**

Although the prevalence of lymphatic filariasis infection has declined since 2000, MDA is still necessary across large populations in Africa and Asia. Our mapped estimates can be used to identify areas where the probability of meeting infection thresholds is low, and when coupled with large uncertainty in the predictions, indicate additional data collection or intervention might be warranted before MDA programmes cease.

**Funding:**

Bill & Melinda Gates Foundation.

## Introduction

Lymphatic filariasis is a parasitic infection caused by the filarial nematodes *Wuchereria bancrofti, Brugia malayi,* and *Brugia timori*.^1^ These parasites are transmitted by members of several mosquito genera, particularly *Anopheles*, *Aedes*, *Culex*, and *Mansonia*, with geographic variation in the identity of dominant vectors.^2^ Long-term infection can cause deterioration of the lymphatic system, characterised by severe swelling of the limbs (lymphoedema) and later elephantiasis or lymphoedema of the scrotum (hydrocele). Community-level transmission of infection can be interrupted^1^ by mass treatment with recommended oral regimens of the antihelmintic medicines albendazole, either alone or with ivermectin, or diethylcarbamazine citrate and albendazole, or a combination of all three, depending on the setting. These medicines are given in mass public health campaigns or, in certain settings, through salt fortification with diethylcarbamazine citrate. Treatment of at least 65% of the total population in endemic areas for at least 5–7 consecutive years through annual or biannual mass drug administration (MDA) is recommended by WHO, to reduce the reservoir of microfilaraemia and antigenaemia among humans, with the ultimate goal of interrupting transmission to eliminate lymphatic filariasis as a public health problem.^3^, ^4^ WHO has recommended guidelines^5^ by which national elimination of lymphatic filariasis as a public health problem can be validated, and national programmes are requested to submit dossiers to document baseline prevalence, programme interventions and monitoring activities, prevalence during surveillance after MDA, and availability of care for people with lymphatic filariasis.

Lymphatic filariasis transmission has been documented throughout Africa, southeast Asia, and the Pacific, as well as in focal areas in the Caribbean, South America, and the Middle East.^6^ Use of population-level vector control or MDA began in the 1950s in India, China, Egypt, and Brazil, followed by implementation across Oceania^7^ from the 1960s to the 1990s. In 1997, the World Health Assembly recognised the goal of global elimination of lymphatic filariasis as a public health problem by 2020 under resolution WHA50.29,^8^ in which national programmes would aim to interrupt transmission and control morbidity. Elimination of lymphatic filariasis as a public health problem was first achieved in China in 2007 and South Korea in 2006.^9^, ^10^ Coordinated efforts between ministries of health, international partners, and the research community under the auspices of the Global Programme to Eliminate Lymphatic Filariasis (GPELF)^11^ have been ongoing since WHO launched the programme in 2000. With the adoption of the London Declaration in 2012, the global community reinforced its commitment to elimination. New milestones and targets for elimination of lymphatic filariasis as a public health problem have been proposed by WHO^12^ in line with 2030 objectives for Sustainable Development Goals.

**Research in context**

**Evidence before this study**

A systematic review of literature was done to identify previous global estimates of lymphatic filariasis infection. The first global estimate of lymphatic filariasis prevalence, published in 1996, estimated 83 million people infected, with an additional 42 million living with hydrocele or lymphoedema. This estimate was then updated in 2000 to account for population growth, suggesting a total of 130 million infections. The most recent global analysis was done for 2013, in which 68 million individuals were estimated to be infected. The 2013 estimate accounted for national-level coverage of mass drug administration, extrapolating from the 2000 estimate. These three estimates all relied on the same lymphatic filariasis prevalence data inputs published between 1953 and 1991. While this series of estimates showed significant reductions in lymphatic filariasis infection over time, they did not use data collected since the inception of the Global Programme to Eliminate Lymphatic Filariasis (GPELF), nor did they account for subnational variation in the distribution of infection.

**Added value of this study**

We compiled a georeferenced lymphatic filariasis infection prevalence dataset from the GPELF, published scientific literature, and ministry of health data, to which we applied model-based geostatistics and time series methods. We estimate 2000–18 global all-age prevalence of lymphatic filariasis infection, with corresponding measures of uncertainty. We also present the posterior probability that various infection thresholds proposed to achieve elimination have been reached. Our model improves upon previous efforts by using a time series perspective not included in earlier global estimates, accounting for subnational variation in baseline endemicity and mass drug administration and insecticide-treated net distribution. This study is also the first to implement diagnostic and age adjustments to account for variability in programmatic data collection over the course of implementation.

**Implications of all the available evidence**

Although lymphatic filariasis prevalence substantially declined from 2000 to 2018, it is likely that not all areas will achieve the GPELF targets by the original goal of 2020. This time series analysis quantifies progress towards elimination since 2000. National programmes and implementing partners can use these geospatial estimates to identify areas that might require additional surveillance or intervention to act on the two components of the global elimination strategy: interrupting transmission and controlling morbidity in affected populations.

From the late 1990s onwards, most national lymphatic filariasis elimination programmes implemented some form of baseline mapping to identify implementation units eligible for MDA, such as districts or counties. Eligibility for MDA was generally determined by infection prevalence of more than 1%, measured by night blood smears to detect microfilaraemia, detection of circulating filarial antigen, or presence of known or suspected filarial lymphoedema and hydrocele cases. Global guidelines for monitoring and evaluation of these programmes were first adopted in 2000,^13^ followed by updates in 2005,^14^ and 2011.^3^ Monitoring of MDA is conducted through periodic sentinel site and spot check surveillance, and current guidelines recommend the Transmission Assessment Survey to determine if implementation units can enter the post-MDA surveillance phase. As of 2018, 21 lymphatic filariasis elimination programmes have begun post-MDA surveillance for all implementation units considered endemic, including 15 that have met validation criteria for having eliminated lymphatic filariasis as a public health problem.^15^ 51 countries or territories with ongoing lymphatic filariasis elimination programmes remain, 15 of which have yet to reach full geographic coverage with MDA as of 2018.^15^

Despite the broad scale of lymphatic filariasis data collection since the inception of the GPELF, previous global infection prevalence estimates relied on older data; estimates for 1996,^6^, ^16^ 2000,^17^ and 2013^17^ were based on data extracted from 118 studies published between 1953 and 1991 for national-level analysis.^16^ Although other studies have employed geostatistical methods in lymphatic filariasis-related research, including estimates of population at risk^18^ and pre-control prevalence,^19^ tests for spatial clustering,^20^ country-level prevalence,^16^, ^21^ and forecasted future prevalence in Africa,^22^, ^23^ no previous analysis has used geospatial methods to estimate time trends in global infection prevalence accounting for subnational variation in covariates associated with lymphatic filariasis transmission. We therefore aimed to estimate the global prevalence of lymphatic filariasis to reflect the progress achieved after two decades of the GPELF and identify areas that might warrant additional programme investment to reach elimination goals by 2030.

## Methods

### Search strategy and selection criteria

We did a systematic review of the published literature to identify lymphatic filariasis prevalence data. We searched PubMed, Web of Science, and Scopus for all articles published between database inception and Oct 14, 2016 (supplemented with a later search for articles published before Oct 24, 2018) with the following keywords: “lymphatic filariasis”, “prevalence”, “incidence”, “mass drug administration”, “coverage”, “lymphoedema”, “hydrocele”, “transmission assessment survey”, and “mapping”. Additional programme monitoring data were obtained from WHO under the auspices of the GPELF and the Expanded Special Project for Elimination of Neglected Tropical Diseases. We compiled data from 1990 onwards in order to model infection prevalence from 2000 to 2018. We included publications that reported prevalence of lymphatic filariasis antigenaemia or microfilaraemia, with sample sizes, location of data collection, and method of diagnosis indicated. We excluded publications that only reported data collected before 1985 or results of case-control studies or qualitative research or that were duplicates of existing data sources. Subnational data were georeferenced to the smallest geographic unit possible, typically at the community or implementation unit level. We extracted the survey year, data type (eg, baseline mapping or Transmission Assessment Survey), site-specific geographic identification information (ie, name of community or district), age range of individuals tested, number tested, number of individuals positive, and diagnostic test used (appendix 2 pp 6–40).

### Geospatial covariates

All statistical analysis was done using R version 3.5.1. Because our modelling framework emphasises prediction, we sought to include a range of covariates that could be associated (even indirectly) with lymphatic filariasis infection, in order to span ecological, demographic, and programmatic determinants of transmission. Covariates were tested to maximise prediction and do not assume a specific causal mechanism. We chose environmental factors, such as elevation, precipitation, and temperature, as well as socioeconomic measures potentially associated with vector-borne diseases, such as Human Development Index^24^ and under-5 mortality.^25^ Population coverage with insecticide-treated nets,^26^, ^27^ indoor residual spraying^28^ (for Africa), and lymphatic filariasis MDA (of any drug regimen) interventions known to reduce transmission, and malaria (*Plasmodium falciparum* and *Plasmodium vivax*) prevalence and incidence were included as proxies for exposure to *Anopheles* spp mosquitoes, because they are vectors of *Plasmodium* spp and *W bancrofti* in some lymphatic filariasis-endemic regions. Antimalarial treatment coverage was included for Africa to account for changes in malaria burden that are unrelated to interventions that affect lymphatic filariasis. Time-variant covariates were extracted to their corresponding model years, with the following exceptions: single-year time lags were applied to some covariates hypothesised to have a lagged temporal relationship with lymphatic filariasis prevalence, and when specific years of data were unavailable, the nearest available year of data was used. Variance Inflation Factor analysis was used to exclude redundant covariates (appendix 2 pp 41–49). The effects of covariates in model fit are presented in appendix 2 (pp 78–81).

### Age and diagnostic adjustment

We used age and diagnostic models to adjust input data before the main analysis, yielding adjusted estimates of all-age (0–94 years) infection prevalence in both sexes combined (male and female), as measured by the immunochromatographic diagnostic test (antigenaemia model) or microfilariae observation (microfilaraemia model). Owing to the introduction and rapid adoption of immunochromatographic test card assessments in the mid-2000s and their higher sensitivity than microfilariae observation, data derived from identification of microfilaraemia by blood microscopy were first adjusted to be comparable with immunochromatographic test estimates of filariasis prevalence (and vice versa for the microfilaraemia model). Prevalence measured in a single age group (such as children in the Transmission Assessment Survey) were adjusted to reflect all-age prevalence. To develop these crosswalk models, we identified peer-reviewed published surveys that reported prevalence of filariasis antigenaemia (immunochromatographic test or Filariasis Test Strip) or microfilaraemia (*W bancrofti* or *Brugia* spp) in multiple age groups in the same study population. The age-dependent relationship between prevalence of microfilaraemia and antigenaemia (as determined by immunochromatographic test) was then calculated using surveys that reported both measures by fitting a logistic regression model (whose outcome measure is the log-odds of infection proportion—ie, cases over sample size), with a basis spline on the ratio of antigenaemia to microfilaraemia prevalence by age, to accommodate potential non-linearity in this relationship. Estimated age-specific ratios were then applied to reported microfilaraemia prevalence data in the geospatial modelling dataset to obtain corresponding estimates of immunochromatographic test prevalence. We estimated age-specific prevalence of infection by fitting a logistic regression model with study population-level fixed effects and a basis spline on age using surveys reporting prevalence of antigenaemia (immunochromatographic test or Filariasis Test Strip) or microfilaraemia for multiple age groups. This estimated prevalence-by-age curve was then compared with each datapoint in the full model-based geostatistics modelling dataset to derive scaling factors, with which the curve was adjusted to yield the corresponding estimates of all-age prevalence. Coefficients for all models used for age or diagnostic adjustment were inferred using maximum likelihood optimisation (appendix 2 pp 50–119).

### Geostatistical analysis

Separate Bayesian geostatistical models were fit for each of the following modelling regions, based on a review of lymphatic filariasis endemicity (appendix 2 pp 13–40): Africa and Yemen (including Madagascar, São Tomé and Príncipe, and Comoros), south Asia (India, Sri Lanka, Nepal, and Bangladesh), southeast Asia, and the island of Hispaniola. For the geostatistical models, we first used an ensemble method^29^ that combines the strengths of different modelling frameworks and can improve predictive performance, fitting three submodels to predict lymphatic filariasis prevalence in each modelling region: generalised additive models (binomial model), generalised boosted models (Poisson model), and lasso regression (empirical logit model). All submodels were fit to covariate values extracted at the year and coordinates of georeferenced datapoints and included country-level fixed effects.

Our full model of lymphatic filariasis infection prevalence consisted of a spatially and temporally explicit generalised linear mixed effects model through integrated nested Laplace approximation,^30^ fit using the R-INLA package,^31^ with a binomial likelihood model and minimally informative priors (appendix 2 p 62). Out-of-sample predictions from the three submodels were used as covariates, with a sum-to-one constraint (effectively incorporating a weighted average of these submodel outputs), and models included country random effects and a nugget variance term to account for very small-scale variation. Residual spatiotemporal variation was modelled as a spatiotemporal Gaussian process. Stochastic partial differential equations were used to model the spatial process, with a Matérn spatial covariance function. Temporal covariance was modelled using a first-order autoregressive function. Predictions were generated at the 5-km^2^ resolution using the in-sample submodel predictions as covariates, with 1000 samples drawn from the joint posterior distributions to account for covariance among model parameters; predictions were summarised by their means and 95% uncertainty interval (UI; generated from the 2·5th and 97·5th percentiles). To assess the contribution of temporal effects, an additional time-stationary (but otherwise identical) geospatial model was fit to each region; the in-sample and out-of-sample behaviour of these time-stationary models was compared with the corresponding spatiotemporal models to select a final model for each region. The posterior probabilities that prevalence was less than 1% or 2% in 2018 were calculated at 5-km^2^ resolution using the 1000 samples; these thresholds represent crucial values for decision making for cessation of MDA in lymphatic filariasis elimination programmes.^3^

Geostatistical methods were not practical for the following locations, owing to small area (<25 km^2^), missing covariate data, or limited georeferenced data: American Samoa, Brazil, Cook Islands, Federated States of Micronesia, Fiji, French Polynesia, Guyana, Kiribati, Maldives, Marshall Islands, New Caledonia, Niue, Palau, Samoa, Tonga, Tuvalu, Vanuatu, and Wallis and Futuna. Instead, Bayesian time series models (first order autoregressive or random walk models) were used to fit annual national prevalence (appendix 2 pp 117–119). Estimation of case totals is described in appendix 2 (pp 64–77). Each geostatistical model was run ten times, each time holding out data from one spatially stratified fold, to generate out-of-sample predictions. Out-of-sample performance was examined by mean bias, mean absolute error, root mean square error, 95% data coverage within prediction intervals, and correlations of observed (held-out data) to predicted values.

### Role of the funding source

The funder of the study had no role in study design, data collection, data analysis, data interpretation, or writing of the report. The corresponding author had full access to all the data in the study and had final responsibility for the decision to submit for publication.

## Results

We used a total of 15 829 georeferenced survey locations to fit our models. 14 927 datapoints were used to fit the geospatial models, of which 13 492 (90·1%) were georeferenced to specific coordinates and 1435 (9·6%) could only be georeferenced to areal units, such as districts or counties. 9942 (66·6%) datapoints reported lymphatic filariasis prevalence by immunochromatographic test, 4120 (27·6%) by microfilaraemia, and 865 (5·8%) by other diagnostic methods (Filariasis Test Strip, Brugia Rapid, etc). An additional 1037 inputs were used to generate estimates of infection prevalence for locations for which model-based geostatistics were infeasible.

**Figure 1:**
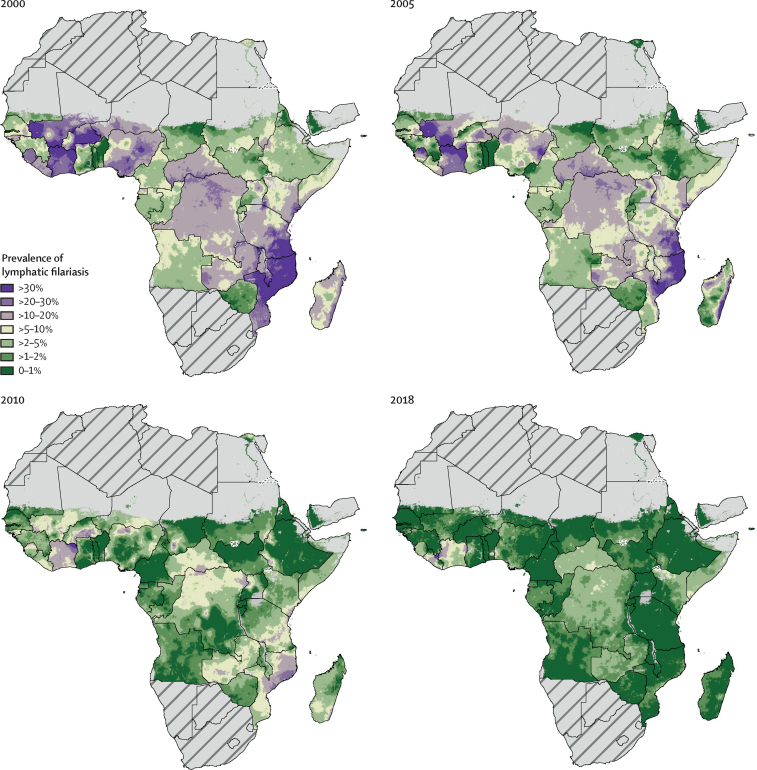
Prevalence of lymphatic filariasis antigenaemia in Africa and Yemen at a 5 km^2^ resolution Mean predictions of lymphatic filariasis antigenaemia (infection) prevalence from the Bayesian geostatistical model for 2000, 2005, 2010, and 2018 in Africa and Yemen, as measured by the immunochromatographic test. Areas for which prevalence exceeded 1% in 2000 would have resulted in the implementation unit (typically a district) qualifying for mass drug administration. Hatch-marks indicate countries for which estimates are not produced. Grey areas are masked on the basis of sparsely populated areas (fewer than 10 people per 1 km^2^ grid cell) and barren landscape classification. Interactive visualisation tool available online.

**Figure 2:**
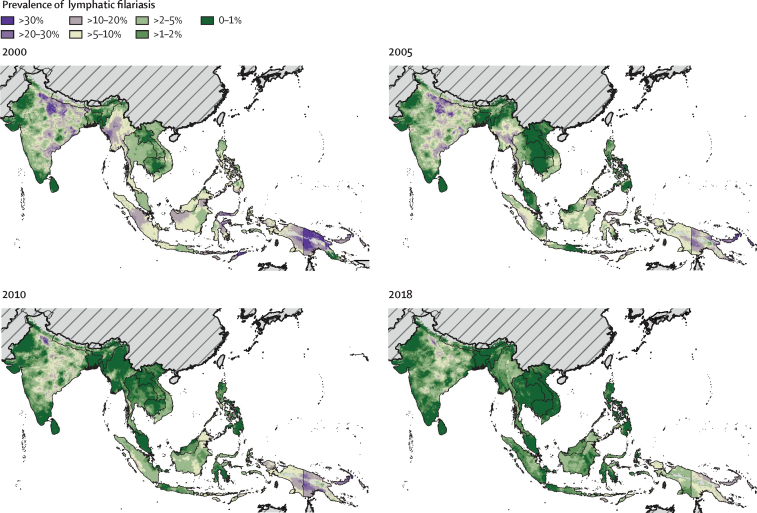
Prevalence of lymphatic filariasis antigenaemia in south and southeast Asia at a 5 km^2^ resolution Mean predictions of lymphatic filariasis antigenaemia (infection) prevalence from the Bayesian geostatistical model for 2000, 2005, 2010, and 2018 in south and southeast Asia, as measured by the immunochromatographic test. Areas for which prevalence exceeded 1% in 2000 would have resulted in the implementation unit (typically a district) qualifying for mass drug administration. Hatch-marks indicate countries for which estimates are not produced. Grey areas are masked on the basis of sparsely populated areas (fewer than 10 people per 1 km^2^ grid cell) and barren landscape classification. Interactive visualisation tool available online.

We estimated a total of 199 million infections in 2000 (95% UI 174–234 million), with totals for WHO regions ranging from 3·1 million (1·6–5·7 million) in the region of the Americas to 107 million (91–134 million) in the South-East Asia region (table). The estimated global burden in 2000 was concentrated in the South-East Asia region, with 52% of the total global number of individuals infected derived from estimates for Bangladesh, India, Indonesia, and Myanmar combined. In Africa, Nigeria, Tanzania, Mozambique, and the Democratic Republic of the Congo comprised around 21% of the 2000 global estimate. The mapped estimated prevalence across Africa for 2000, 2005, 2010, and 2018 (figure 1) shows a general decrease in lymphatic filariasis prevalence over the 19-year period. Globally, the number of individuals infected reduced by 74%, to an estimated 51 million (43–63 million) in 2018 (table). In 2018, the global burden of infection was still mostly concentrated in southeast Asia (figure 2). Although the contribution to the total number of infections is driven by the large population of these countries, countries in coastal west Africa and central Africa, as well as Papua New Guinea, continued to have the highest national prevalence estimates in 2018. Applying different grid-cell-level population thresholds to exclude urban populations from the total estimates of infected individuals resulted in lower regional totals, largely in the South-East Asia region (appendix 2 pp 110–116). Uncertainty of predictions are illustrated in appendix 2 (pp 64–77), which show wider UIs in the earlier time periods and locations for which limited or no data were available, particularly for locations such as the Democratic Republic of the Congo that did not implement baseline mapping until the latter half of our time series. Results of out-of-sample model validation are presented in appendix 2 (pp 92–119).

**Table:** Estimate of individuals with lymphatic filariasis, by WHO region

	**2000**	**2005**	**2010**	**2018**
African region	74 806 947 (59 751 659–93 547 156)	53 285 936 (41 433 633–66 593 897)	22 699 590 (17 650 038–29 487 073)	10 142 514 (6 732 523–15 631 274)
Region of the Americas	3 115 289 (1 557 956–5 667 033)	1 296 451 (686 440–2 270 334)	470 720 (223 958–946 375)	368 629 (153 412–812 803)
Eastern Mediterranean region	3 684 550 (2 148 922–6 804 707)	1 169 399 (397 804–3 694 354)	1 711 945 (750 046–3 718 240)	982 521 (166 924–3 347 885)
South-East Asia region	106 819 113 (90 789 357–134 298 186)	85 737 230 (74 067 271–101 995 083)	50 694 194 (42 897 155–63 174 481)	36 783 583 (30 601 450–46 532 997)
Western Pacific region	8 967 559 (3 165 419—21 686 740)	4 768 730 (2 139 517–11 748 685)	4 242 981 (1 470 866–11 528 780)	2 568 909 (1 042 163–6 234 225)
Total	198 758 026 (174 061 903–234 011 832)	147 319 711 (130 326 019–167 892 148)	80 279 264 (69 142 381–96 315 543)	51 417 924 (42 885 379–63 414 821)

Data are mean (95% uncertainty interval). We did not estimate the number of individuals with lymphatic filariasis for the European region.

In Asia, the largest subnational variations in the model estimates were predominantly in areas in Indonesia, as well as in Papua New Guinea and Myanmar. Districts in the Central African Republic and Côte d’Ivoire were consistently estimated to have among the highest infection prevalence in both 2000 and 2018. Areas of Indonesia and Papua New Guinea represent a large proportion of the lymphatic filariasis infection prevalence in both 2000 and 2018. By contrast, Thailand, Laos, Cambodia, and Vietnam had among the lowest prevalence estimates of the countries we modelled, consistent with the focal nature of transmission in those settings and the achievement of elimination goals. Our results reflect notable progress towards elimination, as documented in settings such as Haiti, Bangladesh, Cambodia, Egypt, Malawi, Thailand, and Togo (Figure 1, Figure 2; appendix 2 pp 64–67). In Africa, the 2018 predictions highlight subnational variation in lymphatic filariasis infection prevalence that persists in west and central Africa. Although subnational predictions suggest potential for lymphatic filariasis transmission in some areas historically considered non-endemic, such as northern Kenya, these estimates have very large uncertainty, because no data from after 1990 were available for inclusion in the model.

**Figure 3:**
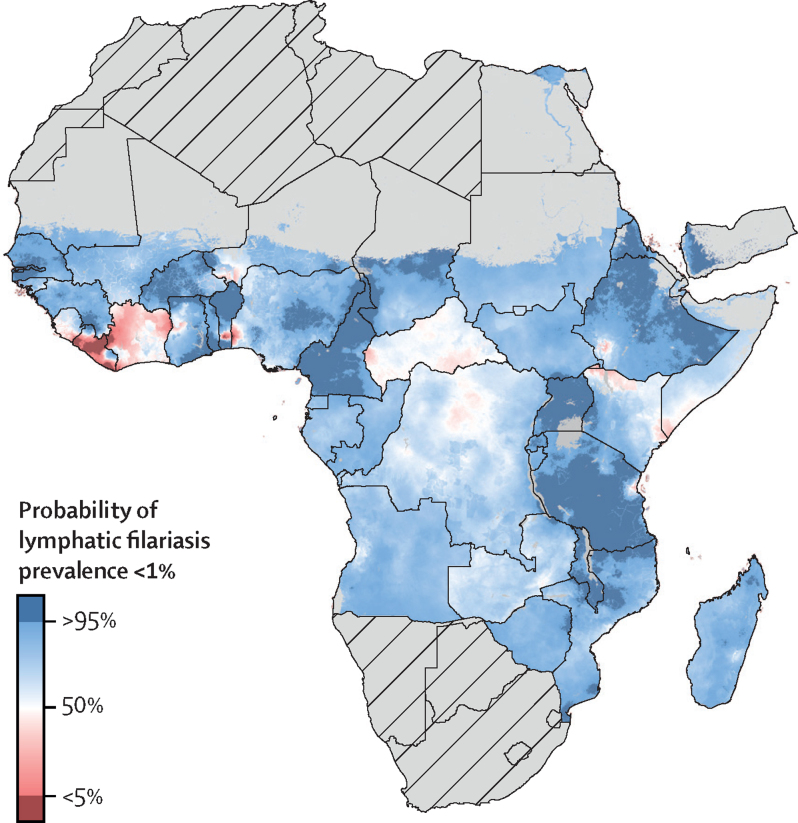
Posterior probability that all-age lymphatic filariasis antigenaemia prevalence was below 1% at a 5 km^2^ resolution in Africa and Yemen, 2018 Mean predictions of posterior probability that all-age lymphatic filariasis antigenaemia (infection) prevalence was below 1% in a given 5 km^2^ grid cell from the Bayesian goestatistcal model for 2018 in Africa and Yemen, as measured by the immunochromatographic test. Hatch-marks indicate countries for which estimates are not produced. Grey areas are masked on the basis of sparsely populated areas (fewer than 10 people per 1 km^2^ grid cell) and barren landscape classification. Interactive visualisation tool available online.

The posterior probability that the prevalence of lymphatic filariasis by immunochromatographic test was less than 1% at the 5-km^2^ resolution varied considerably throughout much of central Africa and coastal west African countries in 2018 (figure 3). Areas in Ghana, Liberia, northern Democratic Republic of the Congo, and Côte d’Ivoire are unlikely to be below this threshold as of 2018. These areas were predicted to be among those with the highest prevalence in 2000, suggesting that higher prevalence in 2000 or more recent programme initiation is predictive of locations that still require MDA. Other regions and results for a 2% threshold are presented in appendix 2 (pp 68–72).

## Discussion

Overall, our results demonstrate the success of the GPELF, reflecting the contribution of donated therapeutics and community-based public health interventions to achieving elimination of a disease that is prevalent among some of the most resource-limited settings in the world. We estimate that more than 198 million individuals were infected globally in 2000, suggesting a greater burden than the around 130 million individuals previously quantified.^17^ The 2018 estimate of around 51 million infected individuals reflects the progress achieved thus far towards the elimination of lymphatic filariasis as a public health problem. Our results are consistent with elimination goals as reported in settings such as Egypt,^32^ Togo,^33^ Malawi,^34^ Cambodia,^35^ Yemen,^36^ and several Pacific Island nations.^37^, ^38^, ^39^, ^40^ Our analysis accounts for subnational variability in environmental covariates and MDA. From the programmatic perspective, the 2018 estimates identify areas for sustained investment, to ensure existing MDA activities continue, and potential areas for confirmatory mapping. National lymphatic filariasis elimination programmes typically follow programme monitoring guidelines that use decision rules on the basis of implementation unit-level data to determine whether MDA should commence, continue, or cease. Given the huge investment in global monitoring for lymphatic filariasis elimination, there is merit to using geospatial analysis for secondary evaluation of these data. Although traditional monitoring approaches are still integral to programme implementation, they depend on field-based data collection for each individual implementation unit to inform decision making, and do not enable national programmes to integrate data from similar settings to update estimates of programme progress. We present a specific use case, in which we calculate the posterior probability that a given prevalence threshold has been met, to identify subnational areas that might pose a threat to achieving elimination goals, which also accounts for uncertainty. In these locations, national programmes might wish to consider prioritising areas for data collection or additional MDA before dismantling programme infrastructure.

Although this global analysis improves upon previous estimates for lymphatic filariasis, data coverage is sparse compared with geospatial analyses of other indicators.^25^ Lymphatic filariasis prevalence data are generally collected for the purpose of MDA implementation, whereas data on indicators such as under-5 mortality monitor progress towards many targets and are therefore common in national household surveys. Data from before 2000 might be subject to bias due to purposive sampling, and data quality has probably improved over time. Global guidelines for lymphatic filariasis data collection recommend small sample sizes for community-level surveys, usually 50–100 individuals, which adds additional uncertainty. We chose not to incorporate lymphatic filariasis endemicity status into our model to avoid circularity. As a result, a few settings (such as northern Kenya, southern Cambodia, or central Thailand) are known to be non-endemic, for which national programmes might not have implemented recent data collection activities or published any data, and our UIs reflect this. Mean estimates of infection prevalence can be greatly affected by extreme predictions among the 1000 iterations, so these results should be interpreted alongside uncertainty, noting that UIs are wide in data-sparse locations. We were unable to account for vector density, parasite aggregation, or annual biting rates, because these covariates are not available for all modelling regions. Although we present probabilities that locations have achieved various thresholds of infection, these should not be interpreted as evidence for the elimination of transmission; rather, low probabilities could serve as a signal to target additional supervision or data collection activities and to highlight uncertainty in the mean infection estimates. Our methods for diagnostics and age adjustment relied on evidence from peer-reviewed literature, with a single age and diagnostic adjustment. Detection of antigenaemia often results in higher prevalence compared with infection measured by microfilaraemia, because antigen-based diagnostics detect the presence of the adult worm. In settings where diethylcarbamazine citrate is included in MDA, the partial macrofilaricidal effects of treatment and the lifespan of the worms might result in detection of dead adult worms. We did not account for possible cross-reactivity due to *Loa loa* infection. We were unable to identify sufficient comparisons to model the age pattern after MDA and the pharmacokinetics of drug regimens. It is plausible that age-specific prevalence varies based on location-specific patterns in MDA participation.

Most lymphatic filariasis burden estimates have either relied on national-level analysis or presented the situation before control programmes started.^19^ National-level analyses do not leverage information from detailed geospatial data sources that now exist for a wide range of covariates, nor do they display subnational variation in prevalence. Presentation of data from before control programmes started generates a cross-sectional estimate of the distribution of infection prevalence that does not account for secular trends, population shifts, and temporal variation in initiation of MDA by implementation units to estimate prevalence for specific years. Our geostatistical models allow the breadth of the lymphatic filariasis data landscape to generate predictions for areas that are less data rich and have programmatic implications. This analysis serves both to quantify the gains achieved towards elimination and to identify areas for which additional data collection or intervention might be warranted before MDA programmes cease.

Correspondence to: Dr Elizabeth A Cromwell, Institute for Health Metrics and Evaluation, University of Washington, Seattle, WA 98121, USA eac27@uw.edu

## Data sharing

This study follows the Guidelines for Accurate and Transparent Health Estimates Reporting. The annual mean estimates and UIs produced by this study can be further explored using online visualisation tools. The data sources and code used to generate these estimates, as well as tabular format of mean estimates and UIs are publicly available online at the Global Health Data Exchange (GHDx). Data sources are listed in appendix 2 (pp 12–36); sharing restrictions are detailed in the GHDx record for each citation. Any unrestricted data can be downloaded from the GHDx. All maps presented in this study were generated by the authors using ArcGIS Desktop 10.6, and no permissions are required to publish them.
