
JOURNAL INFORMATION
==============================
NLM Title Abbreviation: Lancet Glob Health
Journal ID: Lancet Glob Health
NLM Journal ID: 101613665

The Lancet. Global Health

EISSN: 2214-109X

ARTICLE INFORMATION
==============================
PMCID: PMC8480278
PMID: 34534485
DOI: 10.1016/S2214-109X(21)00411-3
Article ID: S2214-109X(21)00411-3
Article version: 1
Subjects: Corrections

Correction to Lancet Glob Health 2020; 8: e1186–94

Publication date: 2021 Oct
Electronic publication date: 2021 Sep 14
Volume: 9
Issue: 10
First page: e1371
Last page: e1371
Copyright: © 2021 The Author(s). Published by Elsevier Ltd.
Copyright year: 2021
Copyright holder: Elsevier Ltd
License: This is an open access article under the CC BY license (http://creativecommons.org/licenses/by/4.0/).
License URL: https://creativecommons.org/licenses/by/4.0/
Erratum for: The global distribution of lymphatic filariasis, 2000–18: a geospatial analysis 8 9 19 8 2020 e1186 e1194 The Lancet. Global Health 10.1016/S2214-109X(20)30286-2 PMC7443698 32827480

==============================
Local Burden of Disease 2019 Neglected Tropical Diseases Collaborators. The global distribution of lymphatic filariasis, 2000–18: a geospatial analysis. Lancet Glob Health 2020; 8: e1186–94—In this Article, Akhil Soman ThekkePurakkal should have been included in the Local Burden of Disease 2019 Neglected Tropical Diseases Collaborators group, and in figures 2 and 3, the boundaries for some countries were incorrect. Appendix 1 has been updated. These corrections have been made as of Sept 14, 2021.

