# Supplementary material for: The global distribution of lymphatic filariasis, 2000–18: a geospatial analysis
Source: Lancet Glob Health. 2020 Aug 19;8(9):e1186–94. doi: 10.1016/S2214-109X(20)30286-2 (PMC7443698; doi:10.1016/S2214-109X(20)30286-2)
Supplement: Supplementary appendix [file mmc1.pdf]

THE LANCET
Global Health

Supplementary appendix 1

This appendix formed part of the original submission and has been peer reviewed. We post it as supplied by the authors.

Supplement to: Local Burden of Disease 2019 Neglected Tropical Diseases Collaborators. The global distribution of lymphatic filariasis, 2000–18: a geospatial analysis. *Lancet Glob Health* 2020; **8:** e1186–94.

LBD 2019 Neglected Tropical Diseases Collaborators

Elizabeth A Cromwell, Chris A Schmidt, Kevin T Kwong, David M Pigott, Denise Mupfasoni, Gautam Biswas, Shreya Shirude, Elex Hill, Katie M Donkers, Amir Abdoli, Michael R M Abrigo, Victor Adekanmbi, Olatunji O Adetokunboh Sr, Srividya Adinarayanan, Ehsan Ahmadpour, Muktar Beshir Ahmed, Temesgen Yihunie Akalu, Fahad Mashhour Alanezi, Turki M Alanzi, Cyrus Alinia, Vahid Alipour, Arianna Maever L Amit Sr, Nahla Hamed Anber, Robert Ancuceanu, Zewudu Andualem, Mina Anjomshoa, Fereshteh Ansari, Carl Abelardo T Antonio, Davood Anvari, Seth Christopher Yaw Appiah, Jalal Arabloo, Benjamin F Arnold, Marcel Ausloos, Martin Amogre Ayanore Sr, Alireza Badirzadeh, Atif Amin Baig Jr., Maciej Banach Sr, Adhanom Gebreegziabher Baraki Sr, Till Winfried Bärnighausen, Mohsen Bayati, Krittika Bhattacharyya Sr, Zulfiqar A Bhutta, Ali Bijani, Donal Bisanzio, Moses John Bockarie, Somayeh Bohlouli, Mehdi Bohluli, Zahid A Butt, Jorge Cano, Felix Carvalho, Vijay Kumar Chattu, Ali Reza Chavshin, Natalie Maria Cormier, Giovanni Damiani, Lalit Dandona, Rakhi Dandona, Aso Mohammad Darwesh, Ahmad Daryani, Aditya Prasad Dash, Kebede Deribe, Aniruddha Deshpande, Blen Kassahun Dessu, Meghnath Dhimal, Mostafa Dianatinasab, Daniel Diaz, Hoa Thi Do, Lucas Earl, Maha El Tantawi, Anwar Faraj, Nazir Fattahi, Eduarda Fernandes, Florian Fischer, Nataliya A Foigt, Masoud Foroutan, Yuming Guo, Gessessew Bugssa Hailu, Ahmed I Hasaballah, Hadi Hassankhani, Claudiu Herteliu, Hagos Degefa de Hidru, Michael K Hole, Julia Hon, Naznin Hossain, Mehdi Hosseinzadeh, Mowafa Househ, Ayesha Humayun, Olayinka Stephen Ilesanmi, Irena M Ilic, Milena D Ilic, Usman Iqbal, Seyed Sina Naghibi Irvani, M Mofizul Islam, Ravi Prakash Jha, John S Ji, Kimberly B Johnson, Jacek Jerzy Jozwiak, Ali Kabir, Leila R Kalankesh, Rohollah Kalhor, Behzad Karami Matin, André Karch, Salah Eddin Karimi, Amir Kasaeian, Gbenga A Kayode, Ali Kazemi Karyani, Abraham Getachew Kelbore, Morteza Abdullatif Khafaie, Rovshan Khalilov, Junaid Khan, Khaled Khatab, Mona M Khater, Mohammad Taghi Khodayari, Neda Kianipour, Yun Jin Kim, Damaris K Kinyoki, G Anil Kumar, Dian Kusuma, Carlo La Vecchia, Van Charles Lansingh, Paul H Lee, Kate E LeGrand, Aubrey J Levine, Shanshan Li, Shokofeh Maleki, Mohammad Ali Mansournia, Francisco Rogerlândio Martins-Melo, Benjamin Ballard Massenburg, Benjamin K Mayala, Wahengbam Bigyananda Meitei, Walter Mendoza, Desalegn Tadese Mengistu, Seid Tiku Mereta, Tomislav Mestrovic, Kebadnew Mulatu Mihretie, Molly K Miller-Petrie, Abdollah Mohammadian-Hafshejani, Shafiu Mohammed, Ali H Mokdad, Masoud Moradi, Rahmatollah Moradzadeh, Paula Moraga, Shane Douglas Morrison, Jonathan F Mosser, Seyyed Meysam Mousavi, Sandra B Munro, Saravanan Muthupandian, Upendo J mwingira, Mehdi Naderi, Ahamarshan Jayaraman Nagarajan, Gurudatta Naik, Ionut Negoi, Trang Huyen Nguyen, Huong Lan Thi Nguyen, Andrew T Olagunju, Ahmed Omar Bali, Osayomwanbo Osarenotor, Frank B Osei, Deepak Kumar Pasupula, Meghdad Pirsaheb, Hadi Pourjafar, Priya Rathi, David Laith Rawaf, Salman Rawaf, Reza Rawassizadeh, Robert C Reiner Jr, Melese Abate Reta, Aziz Rezapour, Ana Isabel

Ribeiro, Ali Rostami, Shanmugavelu Sabesan, Ehsan Sadeghi, S Mohammad Sajadi, Abdallah M Samy, Benn Sartorius, Lauren E Schaeffer, Masood Ali Shaikh, Kiomars Sharafi, Zeinab Sharafi, Hamid Sharifi, Kenji Shibuya, Jae Il Shin, Amin Soheili, Shahin Soltani, Adel Spotin, Wilma A Stolk, Berhe Etsay Tesfay, Roman Topor-Madry, Khanh Bao Tran, Bach Xuan Tran, Irfan Ullah, Bhaskaran Unnikrishnan, Yasser Vasseghian, Natalie V S Vinkeles Melchers, Francesco S Violante, Tomohide Yamada, Sanni Yaya, Vahid Yazdi-Feyzabadi, Paul Yip, Naohiro Yonemoto, Leila Zaki, Sojib Bin Zaman, Maryam Zamanian, Alireza Zangeneh, Zhi-Jiang Zhang, Yunquan Zhang, Arash Ziapour, Jonathan D King, and Simon I Hay

Affiliations

Institute for Health Metrics and Evaluation (E A Cromwell PhD, C A Schmidt PhD, K T Kwong MPH,D M Pigott PhD, S Shirude MPH, E Hill BA, K M Donkers BS, N M Cormier MPSA, Prof. L Dandona MD, Prof. R Dandona PhD, A Deshpande MPH, L Earl, J Hon MLS, K B Johnson MS, D K Kinyoki PhD, K E LeGrand MPH, A J Levine MSPH, B K Mayala PhD, M K Miller-Petrie MSc, Prof. A H Mokdad PhD, J F Mosser MD, S B Munro PhD, R C Reiner Jr PhD, L E Schaeffer MS, Prof. S I Hay FMedSci), Department of Health Metrics Sciences, School of Medicine (E A Cromwell PhD, D M Pigott PhD, D K Kinyoki PhD, Prof. A H Mokdad PhD, R C Reiner Jr PhD, Prof. B Sartorius PhD, Prof. S I Hay FMedSci), Division of Plastic and Reconstructive Surgery (B B Massenburg MD), Division of Plastic and Reconstructive Surgery (S D Morrison MD), University of Washington, Seattle, WA, USA; Department of Control of Neglected Tropical Diseases (D Mupfasoni MD, G Biswas MD, J D King PhD), World Health Organization, Geneva, Switzerland; Department of Parasitology and Mycology (A Abdoli PhD), Jahrom University of Medical Sciences, Jahrom, Iran; Department of Research (M R M Abrigo PhD), Philippine Institute for Development Studies, Quezon City, Philippines; Population Health Sciences (V Adekanmbi PhD), King's College London, London, England; Centre of Excellence for Epidemiological Modelling and Analysis (O O Adetokunboh Sr. PhD), Stellenbosch University, Stellenbosch, South Africa; Department of Global Health (O O Adetokunboh Sr. PhD), Stellenbosch University, Cape Town, South Africa; Vector Control Research Centre (S Adinarayanan PhD, S Sabesan PhD), Indian Council of Medical Research, Puducherry, India; Infectious and Tropical Diseases Research Center (E Ahmadpour PhD), Research Center for Evidence Based Medicine (F Ansari PhD), School of Nursing and Midwifery (H Hassankhani PhD), Health Services Management Research Center (L R Kalankesh PhD), Social Determinants of Health Research Center (S Karimi PhD), Department of Public Health (M Khodayari PhD), Department of Parasitology and Mycology (A Spotin PhD), Tabriz University of Medical Sciences, Tabriz, Iran (E Ahmadpour PhD); Department of Epidemiology (M B

Ahmed MPH), Department of Environmental Health Sciences and Technology (S Mereta PhD), Jimma University, Jimma, Ethiopia; Australian Center for Precision Health (M B Ahmed MPH), University of South Australia, Adelaide, SA, Australia; Department of Epidemiology and Biostatistics (T Y Akalu MPH, A G Baraki Sr. MPH), The Department of Environmental Health and Occupational Health and Safety (Z Andualem MSc), University of Gondar, Gondar, Ethiopia; Health Information Management and Technology Department (T M Alanzi PhD), Imam Abdulrahman Bin Faisal University, Dammam, Saudi Arabia (F M Alanezi PhD); Department of Health Care Management and Economics (C Alinia PhD), Department of Medical Entomology and Vector Control (A Chavshin PhD), Urmia University of Medical Science, Urmia, Iran; Health Management and Economics Research Center (V Alipour PhD, J Arabloo PhD, A Rezapour PhD), Health Economics Department (V Alipour PhD), Department of Parasitology and Mycology (A Badirzadeh PhD), Minimally Invasive Surgery Research Center (A Kabir MD), Pars Advanced and Minimally Invasive Medical Manners Research Center (A Kasaeian PhD), Iran University of Medical Sciences, Tehran, Iran (A Badirzadeh PhD); Department of Epidemiology and Biostatistics (A L Amit Sr. BS), Department of Health Policy and Administration (C T Antonio MD), University of the Philippines Manila, Manila, Philippines; School of Public Health (A L Amit Sr. BS), Johns Hopkins University, Baltimore, MD, USA; Faculty of Medicine (N H Anber DrPH), Mansoura University, Mansoura, Egypt (N H Anber DrPH); Pharmacy Department (Prof. R Ancuceanu PhD), Department of General Surgery (I Negoi PhD), Carol Davila University of Medicine and Pharmacy, Bucharest, Romania; Social Determinants of Health Research Center (M Anjomshoa PhD), Rafsanjan University of Medical Sciences, Rafsanjan, Iran; Razi Vaccine and Serum Research Institute (F Ansari PhD), Agricultural Research, Education, and Extension Organization (AREEO), Tehran, Iran; Department of Applied Social Sciences (C T Antonio MD), School of Nursing (P H Lee PhD), Hong Kong Polytechnic University, Hong Kong, China; Department of Parasitology (D Anvari PhD), Toxoplasmosis Research Center (Prof. A Daryani PhD), Mazandaran University of Medical Sciences, Sari, Iran; Department of Parasitology (D Anvari PhD), Iranshahr University of Medical Sciences, Iranshahr, Iran; Department of Sociology and Social Work (S Appiah PhD), Kwame Nkrumah University of Science and Technology, Kumasi, Ghana; Center for International Health (S Appiah PhD), Ludwig Maximilians University, Munich, Germany; Department of Ophthalmology (B F Arnold PhD), University of California San Francisco, San Francisco, CA, USA; School of Business (Prof. M Ausloos PhD), University of Leicester, Leicester, UK; Department of Statistics & Econometrics (Prof. M Ausloos PhD), Department of Statistics and Econometrics (Prof. C Herteliu PhD), Bucharest University of Economic Studies, Bucharest, Romania; Department of Health Policy Planning and Management (M A Ayanore Sr. PhD), University of Health and Allied Sciences, Ho, Ghana; Unit of Biochemistry (A A Baig Jr. PhD), School of Health Sciences (A A Baig Jr. PhD), Sultan Zainal Abidin University, Kuala Terengganu, Malaysia; Department

of Hypertension (Prof. M Banach Sr. PhD), Medical University of Lodz, Lodz, Poland; Polish Mothers' Memorial Hospital Research Institute, Lodz, Poland (Prof. M Banach Sr. PhD); Heidelberg Institute of Global Health (HIGH) (Prof. T W Bärnighausen MD, S Mohammed PhD), Heidelberg University, Heidelberg, Germany; T.H. Chan School of Public Health (Prof. T W Bärnighausen MD), Harvard University, Boston, MA, USA; Health Human Resources Research Center (M Bayati PhD), Department of Epidemiology (M Dianatinasab MSc), Shiraz University of Medical Sciences, Shiraz, Iran; Department of Statistical and Computational Genomics (K Bhattacharyya Sr. MSc), National Institute of Biomedical Genomics, Kalyani, India; Department of Statistics (K Bhattacharyya Sr. MSc), University of Calcutta, Kolkata, India; Centre for Global Child Health (Prof. Z A Bhutta PhD), Department of Medicine (V Chattu MD), University of Toronto, Toronto, ON, Canada; Centre of Excellence in Women & Child Health (Prof. Z A Bhutta PhD), Aga Khan University, Karachi, Pakistan; Social Determinants of Health Research Center (A Bijani PhD), Infectious Diseases and Tropical Medicine Research Center (A Rostami PhD), Babol University of Medical Sciences, Babol, Iran; Global Health Division (D Bisanzio PhD), Research Triangle Institute International, Research Triangle Park, NC, USA; School of Medicine (D Bisanzio PhD), University of Nottingham, Nottingham, UK; European & Developing Countries Clinical Trials Partnership, Cape Town, South Africa (Prof. M J Bockarie MSc); Department of Medicine (Prof. M J Bockarie MSc), University of Cape Town, Cape Town, South Africa; Department of Veterinary Medicine (S Bohlouli PhD), Islamic Azad University, kermanshah, Iran; Department of Computer Science and Information Technology (M Bohluli PhD), Institute for Advanced Studies in Basic Sciences, Zanjan, Iran; Department of Research and Innovation (M Bohluli PhD), Petanux Research GmBH, Bonn, Germany; School of Public Health and Health Systems (Z A Butt PhD), University of Waterloo, Waterloo, ON, Canada; Al Shifa School of Public Health (Z A Butt PhD), Al Shifa Trust Eye Hospital, Rawalpindi, Pakistan; Department of Disease Control (J Cano PhD), Faculty of Infectious and Tropical Diseases (Prof. B Sartorius PhD), London School of Hygiene & Tropical Medicine, London, UK; Research Unit on Applied Molecular Biosciences (UCIBIO) (Prof. F Carvalho PhD), Associated Laboratory for Green Chemistry (LAQV) (Prof. E Fernandes PhD), EPIUnit - Public Health Institute University Porto (ISPUP) (A Ribeiro PhD), University of Porto, Porto, Portugal; Clinical Dermatology, IRCCS Istituto Ortopedico Galeazzi (G Damiani MD), Department of Clinical Sciences and Community Health (Prof. C La Vecchia MD), University of Milan, Milan, Italy; Department of Dermatology (G Damiani MD), Case Western Reserve University, Cleveland, OH, USA; Public Health Foundation of India, Gurugram, India (Prof. L Dandona MD, Prof. R Dandona PhD, G Kumar PhD); Department of Health Metrics Science (Prof. R Dandona PhD), University of Washington, Seattle, WA; Department of Information Technology (A M Darwesh PhD), Department of Computer Science (M Hosseinzadeh PhD), University of Human Development, Sulaymaniyah, Iraq; Central University Tami Nadu, Thiruvarur,

India (Prof. A P Dash DSc); Wellcome Trust Brighton and Sussex Centre for Global Health Research (K Deribe PhD), Brighton and Sussex Medical School, Brighton, UK; School of Public Health (K Deribe PhD), Addis Ababa University, Addis Ababa, Ethiopia; Department of Anesthesia (B Dessu MSc), Department of Dermatology (A G Kelbore MSc), Wolaita Sodo University, Wolaita Sodo, Ethiopia; Health Research Section (M Dhimal PhD), Nepal Health Research Council, Kathmandu, Nepal; Department of Epidemiology and Biostatistics (M Dianatinasab MSc), Shahroud University of Medical Sciences, Shahroud, Iran; Center of Complexity Sciences (Prof. D Diaz PhD), National Autonomous University of Mexico, Mexico City, Mexico; Faculty of Veterinary Medicine and Zootechnics (Prof. D Diaz PhD), Autonomous University of Sinaloa, Culiacan Rosales, Mexico; Center of Excellence in Public Health Nutrition (H T Do MD), Center of Excellence in Behavioral Medicine (T H Nguyen BMedSc), Nguyen Tat Thanh University, Ho Chi Minh City, Vietnam; Pediatric Dentistry and Dental Public Health Department (Prof. M El Tantawi PhD), Alexandria University, Alexandria, Egypt; Department of Political Science (Prof. A Faraj PhD), Diplomacy and Public Relations Department (A Omar Bali PhD), University of Human Development, Sulaimaniyah, Iraq; Research Center for Environmental Determinants of Health (N Fattahi PhD, Prof. B Karami Matin PhD, A Kazemi Karyani PhD, M Moradi PhD, Prof. M Pirsaheb PhD, Prof. E Sadeghi PhD, K Sharafi PhD, S Soltani PhD, Y Vasseghian PhD), Department of Public Health (A Kazemi Karyani PhD, N Kianipour MA), Clinical Research Development Center (S Maleki MSc, M Naderi PhD), Social Development and Health Promotion Research Center (A Zangeneh MSc), Department of Health Education and Health Promotion (A Ziapour PhD), Kermanshah University of Medical Sciences, Kermanshah, Iran (M Moradi PhD); Institute of Gerontological Health Services and Nursing Research (F Fischer PhD), Ravensburg-Weingarten University of Applied Sciences, Weingarten, Germany; Institute of Gerontology (N A Foigt PhD), National Academy of Medical Sciences of Ukraine, Kyiv, Ukraine; Department of Medical Parasitology (M Foroutan PhD), Abadan Faculty of Medical Sciences, Abadan, Iran; Department of Epidemiology and Preventive Medicine (Prof. Y Guo PhD), School of Public Health and Preventive Medicine (S Li PhD), The School of Clinical Sciences at Monash Health (S Zaman MPH), Monash University, Melbourne, VIC, Australia; Department of Epidemiology (Prof. Y Guo PhD), Binzhou Medical University, Yantai City, China; Department of Medical Parasitology and Entomology (G B Hailu MSc), School of Medicine (D T Mengistu MSc), Department of Microbiology and Immunology (S Muthupandian PhD), Mekelle University, Mekelle, Ethiopia; Department of Zoology and Entomology (A I Hasaballah PhD), Al Azhar University, Cairo, Egypt; Independent Consultant, Tabriz, Iran (H Hassankhani PhD); School of Business (Prof. C Herteliu PhD), London South Bank University, London, UK; Department of Public Health (H D d Hidru MPH, B E Tesfay MPH), Adigrat University, Adigrat, Ethiopia; Department of Pediatrics (M K Hole MD), University of Texas Austin, Austin, TX, USA; Department of Pharmacology (N Hossain

MPhil, N Hossain MPhil), Bangladesh Industrial Gases Limited, Tangail, Bangladesh; Institute of Research and Development (M Hosseinzadeh PhD), Duy Tan University, Da Nang, Vietnam; College of Science and Engineering (Prof. M Househ PhD), Hamad Bin Khalifa University, Doha, Qatar; Department of Public Health and Community Medicine (Prof. A Humayun PhD), Shaikh Khalifa Bin Zayed Al-Nahyan Medical College, Lahore, Pakistan; Department of Community Medicine (O S Ilesanmi PhD), University of Ibadan, Ibadan, Nigeria; Department of Community Medicine (O S Ilesanmi PhD), University College Hospital, Ibadan, Ibadan, Nigeria; Faculty of Medicine (I M Ilic PhD), University of Belgrade, Belgrade, Serbia; Department of Epidemiology (Prof. M D Ilic PhD), University of Kragujevac, Kragujevac, Serbia; College of Public Health (U Iqbal PhD), Taipei Medical University, Taipei, Taiwan; Research Institute for Endocrine Sciences (S N Irvani MD), Shahid Beheshti University of Medical Sciences, Tehran, Iran; School of Psychology and Public Health (M Islam PhD), La Trobe University, Bundoora, Melbourne, VIC, Australia; Department of Community Medicine (R P Jha MSc), Dr. Baba Saheb Ambedkar Medical College & Hospital, Delhi, India; Department of Community Medicine (R P Jha MSc), Banaras Hindu University, Varanasi, India; Environmental Research Center (J S Ji DSc), Duke Kunshan University, Kunshan, China; Nicholas School of the Environment (J S Ji DSc), Duke University, Durham, NC, USA; Department of Family Medicine and Public Health (J J Jozwiak PhD), University of Opole, Opole, Poland; Institute for Prevention of Non-communicable Diseases (R Kalhor PhD), Health Services Management Department (R Kalhor PhD), Qazvin University of Medical Sciences, Qazvin, Iran; Institute for Epidemiology and Social Medicine (A Karch MD), University of Münster, Münster, Germany; Hematology, Oncology and Stem Cell Transplantation Research Center (A Kasaeian PhD), Department of Epidemiology and Biostatistics (M Mansournia PhD), Department of Health Policy, Management, and Economics (S Mousavi PhD), Tehran University of Medical Sciences, Tehran, Iran; International Research Center of Excellence (G A Kayode PhD), Institute of Human Virology Nigeria, Abuja, Nigeria; Julius Centre for Health Sciences and Primary Care (G A Kayode PhD), Utrecht University, Utrecht, Netherlands; Social Determinants of Health Research Center (M A Khafaie PhD), Ahvaz Jundishapur University of Medical Sciences, Ahvaz, Iran; Department of Biophysics and Molecular Biology (Prof. R Khalilov PhD), Baku State University, Baku, Azerbaijan; Institute of Radiation Problems (Prof. R Khalilov PhD), Azerbaijan National Academy of Sciences, Baku, Azerbaijan; Department of Population Studies (J Khan MPhil), Department of Public Health and Mortality Studies (W B Meitei MSc), International Institute for Population Sciences, Mumbai, India; Faculty of Health and Wellbeing (K Khatab PhD), Sheffield Hallam University, Sheffield, UK; College of Arts and Sciences (K Khatab PhD), Ohio University, Zanesville, OH, USA; Department of Medical Parasitology (M M Khater MD), Cairo University, Cairo, Egypt; Department of Public Health (M Khodayari PhD), Department of Nutrition and Food Sciences (H Pourjafar PhD), Maragheh University of

Medical Sciences, Maragheh, Iran; School of Traditional Chinese Medicine (Y Kim PhD), Xiamen University Malaysia, Sepang, Malaysia; Imperial College Business School (D Kusuma DSc), WHO Collaborating Centre for Public Health Education and Training (D L Rawaf MD), Department of Primary Care and Public Health (Prof. S Rawaf MD), Imperial College London, London, UK; Faculty of Public Health (D Kusuma DSc), University of Indonesia, Depok, Indonesia; Medical Director (Prof. V C Lansingh PhD), HelpMeSee, New York, NY, USA; General Director (Prof. V C Lansingh PhD), Mexican Institute of Ophthalmology, Queretaro, Mexico; Campus Caucaia (F R Martins-Melo PhD), Federal Institute of Education, Science and Technology of Ceará, Caucaia, Brazil; ICF International (B K Mayala PhD), DHS Program, Rockville, MD, USA; Peru Country Office (W Mendoza MD), United Nations Population Fund (UNFPA), Lima, Peru; Clinical Microbiology and Parasitology Unit (T Mestrovic PhD), Dr. Zora Profozic Polyclinic, Zagreb, Croatia; University Centre Varazdin (T Mestrovic PhD), University North, Varazdin, Croatia; Department of Epidemiology and Biostatistics (K M Mihretie MPH), Bahir Dar University, Bahir Dar, Ethiopia; Department of Epidemiology and Biostatistics (A Mohammadian-Hafshejani PhD), Shahrekord University of Medical Sciences, Shahrekord, Iran; Health Systems and Policy Research Unit (S Mohammed PhD), Ahmadu Bello University, Zaria, Nigeria; Department of Epidemiology (R Moradzadeh PhD, M Zamanian PhD), Arak University of Medical Sciences, Arak, Iran; Department of Mathematical Sciences (P Moraga PhD), University of Bath, Bath, UK; IDG (U J mwingira PhD), Research Triangle Institute International, Washington DC, USA; Nationa Medical Institute for Medical research -NIMR (U J mwingira PhD), National Institutes of Health, Dar es salaam, Tanzania; Research and Analytics Department (A J Nagarajan MTech), Initiative for Financing Health and Human Development, Chennai, India; Department of Research and Analytics (A J Nagarajan MTech), Bioinsilico Technologies, Chennai, India; Comprehensive Cancer Center (G Naik MPH), University of Alabama at Birmingham, Birmingham, AL, USA; Department of General Surgery (I Negoi PhD), Emergency Hospital of Bucharest, Bucharest, Romania; Institute for Global Health Innovations (H L T Nguyen MPH), Duy Tan University, Hanoi, Vietnam; Department of Psychiatry and Behavioural Neurosciences (A T Olagunju MD), McMaster University, Hamilton, ON, Canada; Department of Psychiatry (A T Olagunju MD), University of Lagos, Lagos, Nigeria; Department of Environmental Management and Toxicology (O Osarenotor MSc), University of Benin, Benin City, Nigeria; Faculty of Geo-Information Science and Earth Observation (F B Osei PhD), University of Twente, Enschede, Netherlands; Department of Mathematics and Statistics (F B Osei PhD), University of Energy and Natural Resources, Sunyani, Ghana; Division of General Internal Medicine (D Pasupula MD), University of Pittsburgh Medical Center, Pittsburgh, PA, USA; Dietary Supplements and Probiotic Research Center (H Pourjafar PhD), Alborz University of Medical Sciences, Karaj, Iran; Kasturba Medical College (P Rathi MD), Manipal Academy of Higher Education, Manipal, India; University College London Hospitals,

London, UK (D L Rawaf MD); Academic Public Health England (Prof. S Rawaf MD), Public Health England, London, UK; Department of Computer Science (R Rawassizadeh PhD), Boston University, Boston, MA, USA; Department of Medical Laboratory Science (M A Reta MSc), Woldia University, Woldia, Ethiopia; Department of Medical Microbiology (M A Reta MSc), University of Pretoria, South Africa, South Africa; Department of Phytochemistry (Prof. S Sajadi PhD), Soran University, Soran, Iraq; Department of Nutrition (Prof. S Sajadi PhD), Cihan university-Erbil, Kurdistan Region, Iraq; Department of Entomology (A M Samy PhD), Ain Shams University, Cairo, Egypt; Independent Consultant, Karachi, Pakistan (M A Shaikh MD); Razi Herbal Medicines Research Center (Z Sharafi PhD), Lorestan University of Medical Sciences, Khorramabad, Iran; HIV/STI Surveillance Research Center, and WHO Collaborating Center for HIV Surveillance (Prof. H Sharifi PhD), Health Services Management Research Center (V Yazdi-Feyzabadi PhD), Department of Health Management, Policy, and Economics (V Yazdi-Feyzabadi PhD), Kerman University of Medical Sciences, Kerman, Iran; Institute for Population Health (Prof. K Shibuya MD), King's College London, London, UK; College of Medicine (Prof. J Shin MD), Yonsei University, Seoul, South Korea; Division of Cardiology (Prof. J Shin MD), Emory University, Atlanta, GA, USA; Nursing Care Research Center (A Soheili PhD), Department of Emergency Nursing (A Soheili PhD), Semnan University of Medical Sciences, Semnan, Iran; Department of Public Health (W A Stolk PhD), Control of Infectious Diseases (N V S Vinkeles Melchers MSc), Erasmus University Medical Center, Rotterdam, Netherlands; Institute of Public Health (R Topor-Madry PhD), Jagiellonian University Medical College, Kraków, Poland; Agency for Health Technology Assessment and Tariff System, Warsaw, Poland (R Topor-Madry PhD); Molecular Medicine and Pathology (K B Tran MD), University of Auckland, Auckland, New Zealand; Clinical Hematology and Toxicology (K B Tran MD), Maurice Wilkins Centre, Auckland, New Zealand; Department of Health Economics (B X Tran PhD), Hanoi Medical University, Hanoi, Vietnam; Department of Allied Health Sciences (I Ullah PhD), Iqra National University, Peshawar, Pakistan; Kasturba Medical College (Prof. B Unnikrishnan MD), Manipal Academy of Higher Education, Mangalore, India; Department of Medical and Surgical Sciences (Prof. F S Violante MD), University of Bologna, Bologna, Italy; Occupational Health Unit (Prof. F S Violante MD), Sant'Orsola Malpighi Hospital, Bologna, Italy; Department of Diabetes and Metabolic Diseases (T Yamada MD), University of Tokyo, Tokyo, Japan; School of International Development and Global Studies (Prof. S Yaya PhD), University of Ottawa, Ottawa, ON, Canada; The George Institute for Global Health (Prof. S Yaya PhD), University of Oxford, Oxford, UK; Centre for Suicide Research and Prevention (Prof. P Yip PhD), Department of Social Work and Social Administration (Prof. P Yip PhD), University of Hong Kong, Hong Kong, China; Department of Neuropsychopharmaology (N Yonemoto MPH), National Center of Neurology and Psychiatry, Kodaira, Japan; Department of Public Health (N Yonemoto MPH), Juntendo University, Tokyo, Japan;

Department of Parasitology and Entomology (L Zaki PhD), Tarbiat Modares University, Tehran, Iran; Maternal and Child Health Division (S Zaman MPH), International Centre for Diarrhoeal Disease Research, Bangladesh, Dhaka, Bangladesh; School of Medicine (Z Zhang PhD), Wuhan University, Wuhan, China; School of Public Health (Y Zhang PhD), Hubei Province Key Laboratory of Occupational Hazard Identification and Control (Y Zhang PhD), Wuhan University of Science and Technology, Wuhan, China

Contributors

Research design and study content

Simon Hay, Elizabeth Cromwell, and Jonathan King conceived and planned the study. Elizabeth Cromwell, Elex Hill, Katie Donkers, and Kevin Kwong obtained, extracted, processed, and geo-positioned the study data. Lucas Earl constructed covariate data layers. Chris Schmidt and Kevin Kwong wrote the computer code and designed the statistical analyses. Chris Schmidt carried out the statistical analyses with input from Elizabeth Cromwell, Simon Hay, and Robert C Reiner Jr. Kimberly Johnson, Katie Donkers, and Elex Hill prepared figures. Elizabeth Cromwell wrote the first draft of the manuscript with assistance from Simon Hay and Jonathan King, and all authors contributed to subsequent revisions. All authors provided intellectual inputs into aspects of this study.

Extraction, cleaning, or cataloging data; production of figures and tables

Muktar Ahmed, Nahla Anber, Natalie Cormier, Giovanni Damiani, Ahmad Daryani, Julia Hon, Mowafa Househ, Kimberly Johnson, Rovshan Khalilov, Ali Mokdad, Abdallah M Samy, Chris Schmidt, Yasser Vasseghian

Providing critical feedback on methods or results

Muktar Ahmed, Natalie Cormier, Simon Hay, André Karch, Jonathan Mosser, Nahla Anber, Maciej Banach, Daniel Diaz, morteza abdullatif khafaie, Gurudatta Naik, Ali Reza Chavshin, Kate LeGrand, Michael Hole, Davood Anvari, John Ji, Deepak Kumar Pasupula, Naohiro Yonemoto, Benn Sartorius, Sanni Yaya, Zahid Butt, Andrew T Olagunju, Carl Abelardo Antonio, Vijay Kumar Chattu, Amin Soheili, Zhi-Jiang Zhang, M Mofizul Islam, Benjamin Massenburg, Yunquan Zhang, ProfessorEhsan Sadeghi, Zewudu Andualem, Walter Mendoza, Cyrus Alinia, Mohammad Ali Mansournia, Olatunji Adetokunboh, Berhe Etsay Tesfay, Natalie Vinkeles Melchers, Florian Fischer, Mostafa Dianatinasab, Till Bärnighausen, Jorge Cano, Vahid Yazdi-Feyzabadi, Victor Adekanmbi, kebadnew Mulatu, Jalal Arabloo, Kiomars Sharafi, Moses Bockarie, Gessessew Bugssa, Frank Osei, Milena Ilic, Irena Ilic, Ali Kabir,

Temesgen Yihunie Akalu, Fahad Alanezi, Turki Alanzi, Michael RM Abrigo, Saravanan Muthupandian, Osayomwanbo Osarenotor, Seyed Sina Naghibi Irvani, Maryam Zamanian, Yun Jin Kim, salah eddin karimi, Jacek Jozwiak, Meghdad Pirsaheb, Hadi Pourjafar, Giovanni Damiani, Ionut Negoi, Francisco Rogerlândio Martins-Melo, Mona Khater, Melese Reta, JAE IL SHIN, Naznin Hossain, Kebede Deribe, Tomohide Yamada, Hamid Sharifi, Shokofeh Maleki, Zulfiqar Bhutta, Alireza Badirzadeh, Masoud Foroutan, Vahid Alipour, Masood Ali Shaikh, Ahmad Daryani, Mohsen Bayati, Arianna Maever Amit, Rohollah kalhor, Shanshan Li, S Mohammad Sajadi, Martin Ayanore, Sabesan Shanmugavelu, Ali Bijani, Dash A P, Francesco S Violante, Ana Isabel Ribeiro, Usman iqbal, Yuming Guo, Aziz Rezapour, Leila Zaki, Wahengbam Bigyananda Meitei, Amir Kasaeian, Rahmatollah Moradzadeh, Claudiu Herteliu, Abdollah Mohammadian-Hafshejani, Dian Kusuma, Adhanom Gebreegziabher Baraki, Hoa Thi Do, Bach Tran, Mowafa Househ, Lucas Earl, Paul Yip, Abraham Getachew Kelbore, Blen Kassahun Dessu, Nataliya Foigt, Aniruddha Deshpande, Shafiu Mohammed, Salman Rawaf, Robert Ancuceanu, Benjamin Arnold, Hagos Degefa Hidru, Mehdi Naderi, Jonathan King, Desalegn Tadese, Rovshan Khalilov, Ali Mokdad, Ravi Prakash Jha, Ehsan Ahmadpour, Gbenga Kayode, Ali Rostami, Damaris Kinyoki, Reza Rawassizadeh, Abdallah M Samy, Srividya Adinarayanan, Priya Rathi, Chris Schmidt, Khaled Khatab, Yasser Vasseghian, Junaid Khan, Ayesha Humayun, Ahamarshan Jayaraman Nagarajan, Hadi Hassankhani, Bhaskaran Unnikrishnan, Sojib Bin Zaman, Anwar Faraj, Irfan Ullah, Nazir Fattahi, Wilma Stolk, Mohammad Taghi Khodayari, Lalit Dandona, Rakhi Dandona, Seid Tiku Mereta, Arash Ziapour, Benjamin Mayala, Shahin Soltani, Lauren Schaeffer, Leila R Kalankesh, Donal Bisanzio, Seth Christopher Yaw Appiah, Olayinka Ilesanmi, Meghnath Dhimal, Khanh Bao Tran, Gautam Biswas

Drafting the manuscript or revising it critically for important intellectual content

Muktar Ahmed, Simon Hay, André Karch, Jonathan Mosser, Nahla Anber, Maha El Tantawi, Maciej Banach, Daniel Diaz, Kate LeGrand, Michael Hole, Molly Miller-Petrie, Naohiro Yonemoto, Kenji Shibuya, Sanni Yaya, Shane Morrison, Andrew T Olagunju, Carl Abelardo Antonio, Vijay Kumar Chattu, Amin Soheili, Ahmed Hasaballah, Zhi-Jiang Zhang, Benjamin Massenburg, Walter Mendoza, Cyrus Alinia, Mohammad Ali Mansournia, Olatunji Adetokunboh, Natalie Vinkeles Melchers, Florian Fischer, Carlo La Vecchia, Mostafa Dianatinasab, Till Bärnighausen, Paula Moraga, Vahid Yazdi-Feyzabadi, Victor Adekanmbi, Jalal Arabloo, Gessessew Bugssa, Milena Ilic, Irena Ilic, Ali Kabir, Osayomwanbo Osarenotor, Seyed Sina Naghibi Irvani, Maryam Zamanian, Yun Jin Kim, Jacek Jozwiak, Giovanni Damiani, Felix Carvalho, Ionut Negoi, Francisco Rogerlândio Martins-Melo, Mona Khater, Naznin Hossain, Kebede Deribe, Masoud Foroutan, Masood Ali Shaikh, Ahmad Daryani, Shanshan Li, Martin Ayanore, Tomislav Mestrovic, Francesco S Violante, Ana Isabel Ribeiro, Usman iqbal, Rahmatollah Moradzadeh, Claudiu Herteliu, Abdollah Mohammadian-Hafshejani, Dian Kusuma, Sandra Munro, Hoa

Thi Do, Bach Tran, Mowafa Househ, Paul Lee, Abraham Getachew Kelbore, Blen Kassahun Dessu, Nataliya Foigt, Shafiu Mohammed, Salman Rawaf, Robert Ancuceanu, Jonathan King, Desalegn Tadese, Rovshan Khalilov, Ali Mokdad, Ravi Prakash Jha, Gbenga Kayode, Reza Rawassizadeh, Abdallah M Samy, Srividya Adinarayanan, Chris Schmidt, Khaled Khatab, Zeinab Sharafi, Yasser Vasseghian, Junaid Khan, Ahamarshan Jayaraman Nagarajan, Atif Baig, Bhaskaran Unnikrishnan, Sojib Bin Zaman, Roman Topor-Madry, Anwar Faraj, Irfan Ullah, Wilma Stolk, Arash Ziapour, Lauren Schaeffer, Seyyed Meysam Mousavi, Olayinka Ilesanmi, Meghnath Dhimal

Management of the overall research enterprise (for example, through membership in the Scientific Council)
Muktar Ahmed, Nahla Anber, Kenji Shibuya, Benn Sartorius, Alireza Badirzadeh, Mowafa Househ, Rovshan Khalilov, Ali Mokdad, Yasser Vasseghian, Roman Topor-Madry, Lalit Dandona

Providing data or critical feedback on data sources
Muktar Ahmed, André Karch, Nahla Anber, Maciej Banach, morteza abdullatif khafaie, Michael Hole, Davood Anvari, John Ji, Deepak Kumar Pasupula, Naohiro Yonemoto, Sanni Yaya, Andrew T Olagunju, Vijay Kumar Chattu, Amin Soheili, Ahmed Hasaballah, ProfessorEhsan Sadeghi, Walter Mendoza, Mohammad Ali Mansournia, Olatunji Adetokunboh, Natalie Vinkeles Melchers, Mostafa Dianatinasab, Till Bärnighausen, Jorge Cano, Victor Adekanmbi, Moses Bockarie, Gessessew Bugssa, Michael RM Abrigo, Saravanan Muthupandian, Seyed Sina Naghibi Irvani, Masoud Moradi, salah eddin karimi, Jacek Jozwiak, Hadi Pourjafar, Ionut Negoi, JAE IL SHIN, Kebede Deribe, Masood Ali Shaikh, Ahmad Daryani, S Mohammad Sajadi, Ana Isabel Ribeiro, Usman iqbal, Yuming Guo, Adel Spotin, Amir Kasaeian, Claudiu Herteliu, Neda Kianipour, Alireza Zangeneh, Dian Kusuma, Hoa Thi Do, Bach Tran, Mowafa Househ, Van Lansingh, Blen Kassahun Dessu, Shafiu Mohammed, Kimberly Johnson, Salman Rawaf, Robert Ancuceanu, Jonathan King, Desalegn Tadese, Rovshan Khalilov, Ali Mokdad, Ehsan Ahmadpour, Gbenga Kayode, Ali Rostami, Reza Rawassizadeh, Abdallah M Samy, Priya Rathi, Upendo mwingira, Khaled Khatab, Amir Abdoli, Yasser Vasseghian, Junaid Khan, Ahamarshan Jayaraman Nagarajan, Hadi Hassankhani, Bhaskaran Unnikrishnan, Anwar Faraj, Irfan Ullah, Lalit Dandona, Rakhi Dandona, Arash Ziapour, Benjamin Mayala, Leila R Kalankesh, Lucas Earl, Donal Bisanzio, Seth Christopher Yaw Appiah, Meghnath Dhimal

Development of methods or computational machinery
Nahla Anber, Davood Anvari, Olatunji Adetokunboh, Mostafa Dianatinasab, Giovanni Damiani, Ahmad Daryani, Adel Spotin, Neda Kianipour, Mowafa Househ, Lucas Earl, Rovshan Khalilov, Ali Mokdad,

Abdallah M Samy, Chris Schmidt, Yasser Vasseghian, Mohammad Taghi Khodayari, Arash Ziapour, Lucas Earl, Robert Reiner

Declarations of Interest

Robert Ancuceanu reports he received consultancy and speakers' fees from various pharmaceutical companies. Carl Abelarso Antonio reports personal fees from Johnson & Johnson (Philippines), Inc., outside the submitted work. Jacek Jozwiak reports personal fees from Amgen, personal fees from ALAB, personal fees from Teva, personal fees from Synexus, personal fees from Boehringer Ingelheim, outside the submitted work. Jonathan Mosser reports grants from the Bill and Melinda Gates Foundation, during the conduct of the study. Aniruddha Deshpande reports grants from Bill and Melinda Gates Foundation, during the conduct of the study. Walter Mendoza is Program Analyst in Population and Development at the United Nations Population Fund-UNFPA Country Office in Peru, an institution which does not necessarily endorse this study. All other authors declare no competing interests.

Acknowledgments

Olatunji Adetokunboh would like to acknowledge the South African Department of Science and Innovation, and the National Research Foundation. Till Bärnighausen was supported by the Alexander von Humboldt Foundation through the Alexander von Humboldt Professor award, funded by the German Federal Ministry of Education and Research. Moses Bockarie is supported by the European & Developing Countries Clinical Trials Partnership (EDCTP). Felix Carvalho acknowledges UID/MULTI/04378/2019 and UID/QUI/50006/2019 support with funding from FCT/MCTES through national funds. Kebede Deribe is supported by a grant from the Wellcome Trust [grant number 201900/Z/16/Z] as part of his International Intermediate Fellowship. Marcel Ausloos and Claudiu Herteliu are partially supported by a grant of the Romanian National Authority for Scientific Research and Innovation, CNDS-UEFISCDI, project number PN-III-P4-ID-PCCF-2016-0084. Claudiu Herteliu is partially supported by a grant co-funded by European Fund for Regional Development through the Operational Program for Competitiveness, Project ID P_40_382. Abdallah Samy was supported by a fellowship from the Egyptian Fulbright Mission Program. Sojib Bin Zaman received a scholarship from the Australian Government research training program (RTP) in support of his academic career. Jonathan King would like to acknowledge National LF elimination programmes of the LF endemic countries for programme data reported through WHO Regional and WHO Country Offices.
